# Supplementary material for: Study on the Influence of Host–Guest Structure and Polymer Introduction on the Afterglow Properties of Doped Crystals
Source: Molecules. 2024 Sep 24;29(19):4537. doi: 10.3390/molecules29194537 (PMC11478084; doi:10.3390/molecules29194537)
Supplement: Supplementary file 1 [file molecules-29-04537-s001.zip › molecules-3169441-supplementary.pdf]

Supporting Information

Study on the influence of host-guest structure and polymer introduction on the afterglow properties of doped crystals

Wenhui Feng [1], Zongyong **Lou**[1], **Xiaoqiang Zhao**[1], **Mingming Zhao**[2], **Yaqin Xu**[1], **Yide Gao** [1*]

[1]Department of Thermal Engineering, Hebei Petroleum University of Technology, Cheng De 067000, P. R. China;

[2] School of Chemical Engineering and Technology, Tianjin University, Tianjin 300354, P. R. China;

[*]E-mail: gaoyd106@163.com

Table of contents

1. Characterization data S2
2. Supplementary tables and figures S4
3. References S6

1. Characterization data

TGA. The thermal stability of the samples was determined by measuring the weight loss while heating at a rate of 10 ºC min^{-1} from 25 ºC to 600 ºC under inert nitrogen atmosphere.

DSC. The samples were heated at a rate of 10 ºC min^{-1} from 30 ºC to 300 ºC under inert nitrogen atmosphere.

CV. Cyclic voltammetry is a conventional three-electrode system in which the working electrode is a platinum carbon electrode, the reference electrode is a silver wire (Ag/AgNO3) electrode, and the auxiliary electrode is a platinum wire electrode. Dichloromethane was used as the test solvent in this study, the sample concentration was 5×10-3 mol/L, and the electrolyte was 0.1 mol/L tetrabutylhexafluorophosphate ammonium salt. Before the test, the platinum carbon electrode was ground and polished with aluminum oxide, and the dichloromethane was washed several times and then blown dry. The test solution was deoxygenated with nitrogen gas blowing for 20–30 min. The scanning rate was 30 mV/s and the test was performed at room temperature.

It was measured in dichloromethane that the electric potentials of ferrocene relative to the Ag/AgNO3 electrode and the saturated calomel electrode were 0.26 V and 0.45 V, respectively, so the electric potential of the Ag/AgNO3 electrode relative to the saturated calomel electrode was 0.19 V. The potential of the saturated calomel electrode relative to the standard hydrogen electrode was 0.244 V, and the potential of the standard hydrogen electrode relative to the vacuum was 4.5 V. Therefore, EHOMO = -Eonset - 4.93 eV, ELUMO = -Eonset - 4.93 eV, where Eonset and Eonset are the first oxidation and reduction half-wave potentials, respectively[1-3]. The electrochemical band gap Egel = ELUMO - EHOMO can be calculated from the HOMO and LOMO levels, and the optical band gap Egop = 1241/λonset (UV band edge wavelength) can also be obtained from the band edge of the UV-visible absorption spectrum as a contrast.

TC-SPC. Sample excitation was done with picosecond diode lasers (Horiba Jobin Yvon Instruments) at 321 nm or 375 nm, and the time resolution was ~ 150 ps. The laser pulse energy was ca. 15 pJ and attenuated (often by more than an order of magnitude) to the desired count rate of ca. 1% or less of the excitation frequency. A cooled (ca. -40°C) Hamamatsu MCP- photomultiplier R3809U 51 was used for detection of

single photons, and the signal passed through a discriminator (Ortec 9307) and into a TAC (Ortec 566, 100 ns range used). The electrical trigger signal from the laser was also passed through a discriminator (Tennelec TC454) and on to the TAC (Ortec 566). The TAC output was read by a DAQ-1 MCA computer card using 1024 channels and collected with Horiba Jobin Yvon Data Station 2.5. Measurements were made in reverse mode at 5 MHz and under magic angle polarization. A cut-off filter, GG400 (Excitation at 321 nm) or GG 515 (Excitation at 375 nm), was used to block stray excitation light. A dilute solution of Ludox was used to record the instrument response function without any filter for solution measurements. No monochromator was used, i.e. all wavelengths transmitted by the cut-off filter were collected.

2.Supplementary table and figures

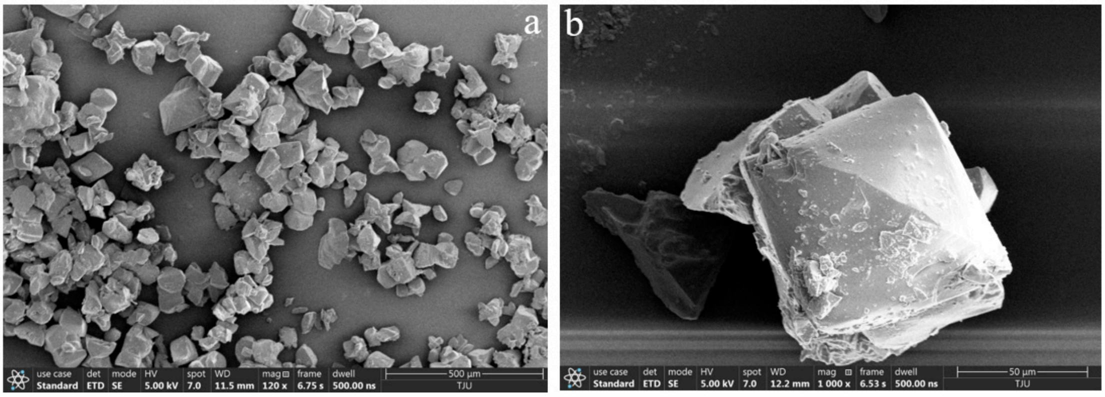

Figure S1. SEM images of MODPA: DDF-O 500 nm (a) and 50 nm (b).

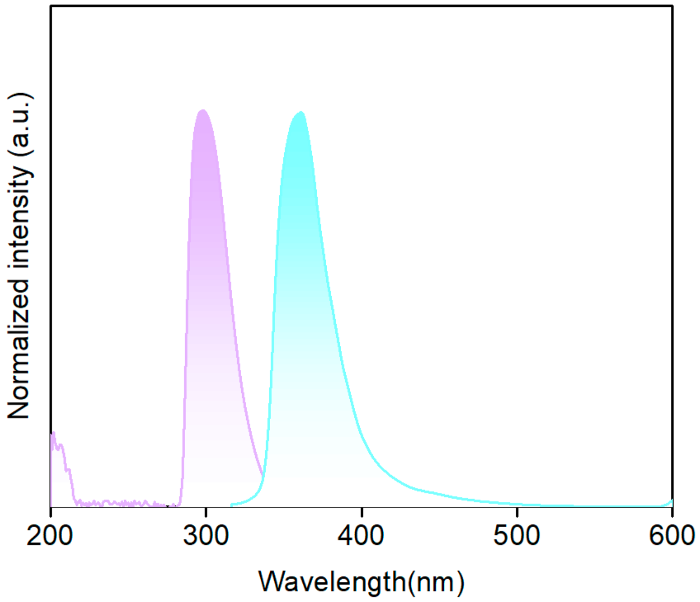

Fig. S2 UV–Vis absorption (purple) and fluorescence (cyan) of the MODPA measured at room temperature under ambient conditions.

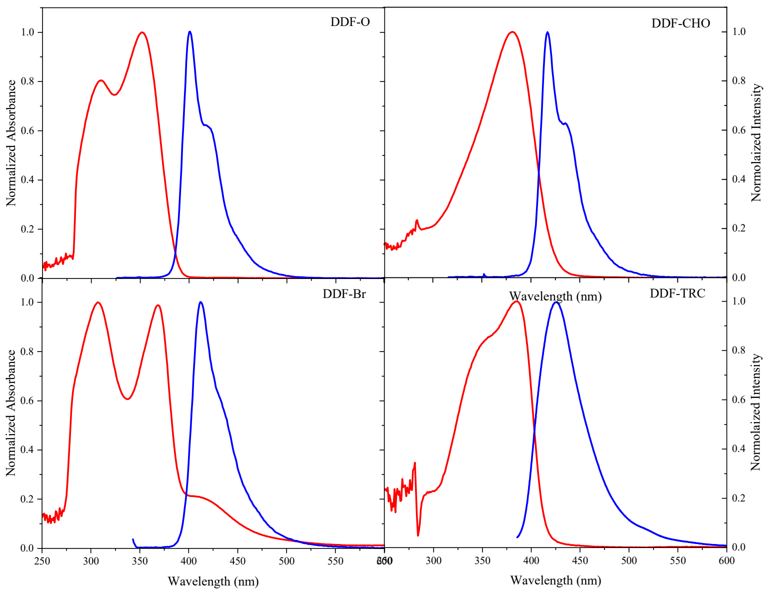

Fig. S3 UV–Vis absorption (red) and fluorescence (blue) of the DDF-O, DDF-CHO, DDF-Br and DDF-TRC measured at room temperature under ambient conditions.

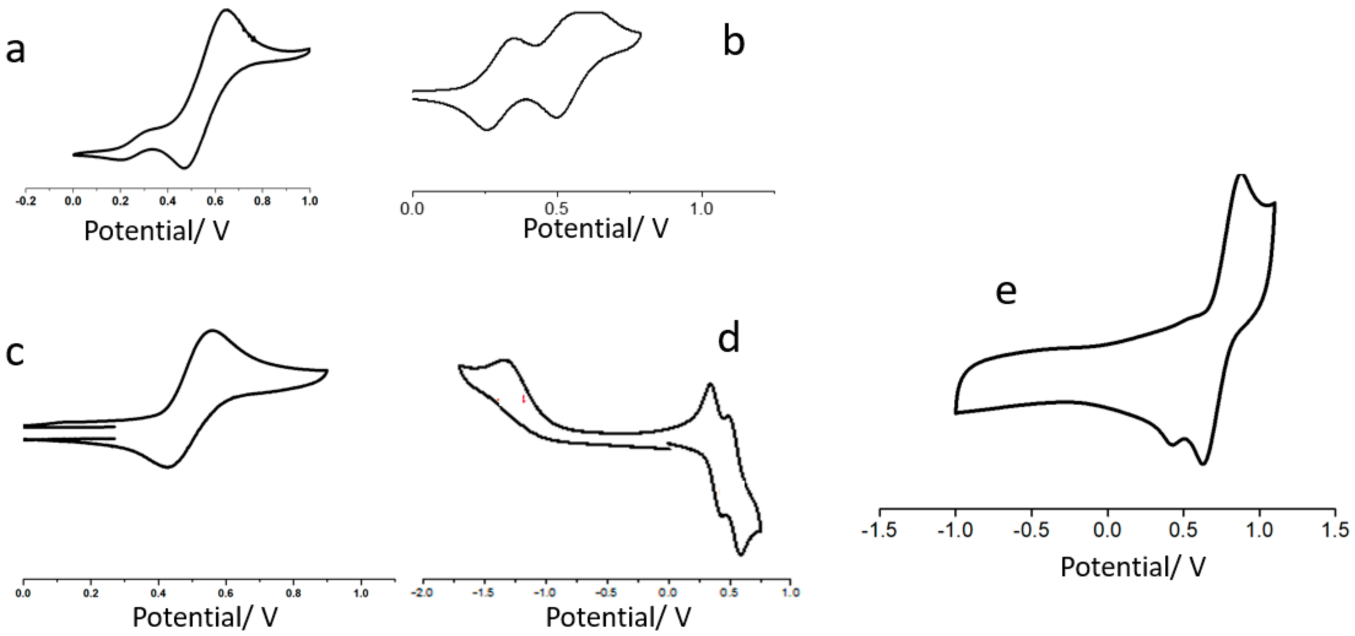

Fig.S4. Electrochemical curves of (a) DDF-O, (b) DDF-CHO, (c) DDF-Br, (d) DDF-TRC and (e) MODPA in dichloromethane vs Ag/Ag+, with the concentration of 5×10^{-3} mol•L^{-1}.

References

[1] Wang T, Weerasinghe K C, Liu D, et al. Ambipolar organic semiconductors with cascades of energy levels for generating long-lived charge separated states: a donor–acceptor1–acceptor2 architectural triarylamine dye. Journal of Materials Chemistry C; 2014, 2(28): 5466-5470.

[2] Song W, Chen X, Wu F, Characterization of band structure of organic polymer material systems. Chem. J. Chinese Universities. 2000; 21, 1422-1426.

[3] Sun H, Liu D, Wang T, Lu T, Li W, Ren S, Hu W, Wang L, and Zhou X. Enhanced Internal Quantum Efficiency in Dye-Sensitized Solar Cells: Effect of Long-Lived Charge-Separated State of Sensitizers. ACS Appl. Mater. Interfaces 2017; 9, 9880–9891.
